# Factors associated with female genital mutilation in Burkina Faso and its policy implications

**DOI:** 10.1186/1475-9276-10-20

**Published:** 2011-05-18

**Authors:** Bue Karmaker, Ngianga-Bakwin Kandala, Donna Chung, Aileen Clarke

**Affiliations:** 1 University of Warwick, Warwick Medical School, Health Science Research Institute, Warwick Evidence, Gibbet Hill, Coventry CV4 7 AL, UK; 2 University of Botswana, Department of population Studies, Gaborone, Botswana; 3 University of Warwick, School of Health and Social Studies, Centre for the Study of Safety and Wellbeing, UK

## Abstract

**Background:**

Female genital mutilation (FGM) usually undertaken between the ages of 1-9 years and is widely practised in some part of Africa and by migrants from African countries in other parts of the world. Laws prohibit FGM in almost every country. FGM can cause immediate complications (pain, bleeding and infection) and delayed complications (sexual, obstetric, psychological problems). Several factors have been associated with an increased likelihood of FGM. In Burkina Faso, the prevalence of FGM appears to have increased in recent years.

**Methods:**

We investigated social, demographic and economic factors associated with FGM in Burkina Faso using the 2003 Demographic Health Survey (DHS). The DHS is a nationally representative cross-sectional survey (multistage stratified random sampling of households) of women of reproductive age (15-49 years). Associations between potential risk factors and the prevalence of FGM were explored using χ2 and t-tests and Mann Whitney U-test as appropriate. Logistic regression modelling was used to investigate social, demographic and economic risk factors associated with FGM.

**Main outcome measures:**

i) whether a woman herself had had FGM; ii) whether she had one or more daughters with FGM.

**Results:**

Data were available on 12,049 women. Response rates by region were at least 90%. Women interviewed were representative of the underlying populations of the different regions of Burkina Faso. Seventy seven percent (9267) of the women interviewed had had FGM. 7336 women had a daughter of whom 2216 (30.2%) had a daughter with FGM and 334 (4.5%) said that they intended that their daughter should have it. Univariate analysis showed that age, religion, wealth, ethnicity, literacy, years of education, household affluence, region and who had responsibility for health care decisions in the household had (RHCD) were all significantly related to the two outcomes (p < 0.01). Multivariate analysis stratified by religion mainly confirmed these findings, however, education is significantly associated with a reduced likelihood of FGM only for Christian women.

**Conclusions and Policy implications:**

Factors associated with FGM are varied and complex. Younger women and those from specific groups and religions are less likely to have had FGM. A higher level of education may be protective for women from certain religions. Policies should capitalize on these findings and religious leaders should be involved in continuing programmes of action.

## Introduction

Female genital cutting or mutilation (FGM) sometimes called female circumcision is a practice which is thought to have existed for thousands of years [1]. It is most commonly practised in countries in northern sub-Saharan Africa; in the Sahel region, in the horn of Africa and Egypt, but it is also found outside Africa e.g. amongst women and families migrating to European countries and the US from these locations [2-6].

It is estimated that world-wide between 100-140 million women are thought to have undergone FGM and 3 million girls annually are thought to be at risk [2]. FGM varies from a more or less ritual and symbolic genital cutting, through to levels of severity which include removal of the clitoris; and/or removal of the labia minora; and/or infibulation (abrasion and stitching together) of the labia majora after which a small hole is left through which urination and sexual activity can occur. This last form requires reopening (and re-closure) for childbirth [1]. FGM is carried out on girls at different ages ranging from babies and toddlers to teenagers. It is frequently carried out in unsterile conditions by traditional practitioners. This is both the result of its traditional form and its illegality in many places means that it is conducted in such conditions. Complications can include immediate urinary and genital tract infection, pain and haemorrhage, complications in childbirth and social, psychological and sexual complications [1]. The public health burdens of FGM include both consequences for women or daughter mortality and ongoing morbidity concerns through their life span.

FGM, a dangerous and potentially life-threatening procedure to which women and girls in many countries are subjected has been viewed as a Human Rights violation in many countries.

More recently, Parliamentarians from all over Africa met in Dakar on 3-4 May, 2010 to push for a continent-wide ban on FGM and calling on the UN to pass a General Assembly resolution appealing for a global FGM ban, as it violates human rights, they argued. Members of parliament from African nations also exchanged lessons learned and actions to take to achieve the ban and resolution. Some 17 African states have banned FGM, among them Burkina Faso, Togo, Senegal and Uganda [7].

Several questions have been asked about FGM: why are harmful practices still widespread, despite the often significant efforts carried out to promote their abandonment? Why do they persist even in areas where attitudes towards them have changed?

FGM is an issue of health equity because in communities where it is practised, FGM is not viewed as a dangerous act and a violation of rights, but as a necessary step to raise a girl properly, to protect her and, in many instances, to make her eligible for marriage.

For policy strategies, multilateral agencies, such as the United Nations Children's Fund in conjunction with the World Health Organization (WHO) and Non Government organisation (NGO) have played a crucial role in advocacy of ending FGM. The NGO such as TOSTAN in Senegal using community led approaches has been proven to be effective in Senegal in eradicating the practice [8]. The authors were pleased to learn from TOSTAN during the recent consultation meeting with UNICEF (second Academic consultation on Social norms, November 18-19, 2010, UNICEF, New York) that TOSTAN is expanding the Senegalese success stories to neighbouring countries in the West African region including Burkina Faso.

Their effort and success stories are also reflected in the achievements of other NGOs in combating the practice using a human rights approach. Kembatta Mentti Gezzimma-Tope (KMG) is an Ethiopia-based indigenous human rights and development NGO that envisions a society where women are free from all forms of discrimination and violence, and where they are able to attain justice, equity, and equality for themselves, their families, and their communities [9].

### Background: Characteristics of the study area

Burkina Faso is an African country located the Western Africa with a population of about 15,757,000 (2009 estimate [10]). Burkina Faso has a population growth rate of about 3.1% percent/year and a total fertility rate of 6.28 children born/woman [11] with a gross national product (GNP) of 183 US$/person/year, around two-thirds of the population live on less than a dollar a day and Burkina Faso is among the 20 least economically developed countries in the world, ranking 161 in term of the Human Development index (HDI) [11].

Life expectancy at birth is 53.7 years with a mean length of schooling (of adults) of 1.3 years. The poverty level or intensity of deprivation is 64.9%. Burkina Faso is a country with a huge gender inequality index. The maternal mortality ratio is estimated at 700 deaths of women per 100,000 live births and the literacy rate (percentage age 15 and over that can read and write) is 21%.

In Burkina Faso the most recent survey shows a small (3%) rise in prevalence of FGM from 73% in 2000 to just over 76% of women in 2003. We used this survey (including the optional additional questions on FGM); to investigate social and household factors associated with FGM in Burkina Faso, in order to consider policy options for next the steps in reducing the prevalence of FGM.

## Methods

### Survey Methods

The Demographic Health Surveys (DHS) are periodic cross sectional health surveys funded by USAID (the U.S. Agency for International Development's) Bureau for Global Health. The DHS includes a number of modules on demographics and household affluence; fertility, reproductive health, maternal and child health; nutrition, and knowledge and practice related to HIV/AIDS [12]. Surveys allow for an optional additional series of questions about FGM [13]. Since the year 2000, when these optional questions were added as part of the DHS, the prevalence of FGM in women in 14 African countries has been found to range between 1% in Cameroon in 2004 to 97% in Egypt in 2000.

The history of the development of the FGM questionnaire has been discussed elsewhere [13]. Briefly, DHS surveys collect data from nationally-representative probability samples of households and from adult women (age 15-49) and men in the sampled household. In general, surveys use a two-stage cluster sampling design, with over-sampling of certain categories of respondents. Response rates tend to vary across sampling domains and sample weights are used to obtain nationally representative estimates of indicators. DHS surveys yield nationally representative estimates of FGM for women age 15 to 49 in the survey countries.

In the countries where the prevalence of FGM is of concern, a module of FGM Questions is added to the women's questionnaire. The questions are designed to generate information on prevalence rates and types of FGM for the women themselves and for their daughters. Respondents' attitudes towards FGM are also collected. Since 2000, UNICEF's Multiple Indicator Cluster Surveys (MICS) have used a similar module to collect information on FGM in selected countries [13]. Both DHS and MICS surveys provide FGM prevalence data. Female respondents are asked if they have ever heard of FGM; then those who have heard of the practice are asked about their own experience of it. The responses to these questions are used to calculate national prevalence rates of FGM [13].

Experts generally assume that women respond truthfully when asked about their own experience. If bias exists in some of the responses, it has not been documented. It is hypothetically possible that some women may say not admit to having undergone FGM in countries where the practice has been forbidden, but no solid evidence of this has been found.

Few empirical studies dealing with FGM in Sub-Saharan Africa have been conducted. Although, there is now an increased amount of literature on the practice of FGM. Most of the literature deals with descriptive statistics and on issues of the origin, types, and justifications for the practice but little has been devoted to its patterns and mediating factors. Freymeyer et. al. 2007 [14] used the 2003 Nigerian DHS to explore attitudes towards FGM in Nigerian. Also using empirical data from the DHS, Kandala et al. 2010 [15] have published work on spatial risk factors for FGM in Nigeria.

The DHS survey methods in Burkina Faso are described in detail elsewhere [12] A nationally representative cross-sectional survey of women of reproductive age (15-49 years) was identified using a stratified random two-stage cluster sample of households by enumeration area, to reflect the rural-urban ratio. 400 (of 11,000) enumeration districts were selected (90 urban and 310 rural). 9 470 households in these districts were selected (2340 urban and 7130 rural) and members of 9097 household successfully interviewed. Data collection was undertaken between June and December 2003 by face to face interview with women aged 15-49 in each selected household and with men aged 15-59 in every third household. Topics covered in the women's questionnaire included demographics: age, region, ethnicity, religion, education, literacy, nutrition, and fertility, family planning, and HIV/AIDS risk factors. Topics covered in the household questionnaire included who in the household had responsibility for health care decisions (RHCD); and a number of household characteristics allowing for a calculation of household affluence using an asset index (access to drinking water, toilet facilities, cooking fuel, consumer items (television, bike/car); wall/flooring material. Households were subsequently divided into quintiles using this asset index.

The module on FGM included questions on whether the woman herself had undergone FGM; and whether if she had daughters they had also undergone the practice (Table 1) or whether she intended that they should.

**Table 1:** Distribution of factors analysed in Female Genital Mutilation (FGM) study in Burkina Faso (DHS 2003)

	Respondent has had FGM N (%)			1 or more daughters has had FGM N (%)		
	**No**	**Yes**	**p-value**	**No**	**Yes**	**p-value**
Age of respondent (years)						
15-24	1401(29.3)	3378(70.7)	<0.001	1126(92.5)	91(7.5)	<0.001
25-34	745(21.3)	2760(78.7)		2158(79.2)	565(20.7)	
35-49	636(16.9)	3129(83.1)		1836(54.1)	1560(45.9)	
Religion						
Catholic	980(33.0)	1986(67.0)	<0.001	1308(78.1)	277(32.6)	<0.001
Protestant	251(38.2)	406(61.8)		306(84.5)	56(15.5)	
Muslim	1106(16.4)	5656(83.6)		2677(64.8)	1455(35.2)	
Traditionalist/Animist	444(26.4)	1238(73.6)		828(71.0)	338(29.0)	
Place of residence						
urban	755(25.3)	2227(74.7)	0.001	1024(72.4)	390(27.6)	0.017
rural	2027(22.4)	7040(77.6)		4096(69.2)	1826(30.8)	
RHCD*						
Respondent alone	267(21.4)	981(78.6)	0.09	573(67.4)	277(32.6)	0.10
Respondent and husband	151(16.7)	755(83.3)		477(68.9)	215(31.1)	
Husband/partner	1451(20.4)	5653(79.6)		3705(69.9)	1594(30.1)	
Other e.g community leader	913(32.7)	1877(67.3)		365(73.7)	130(26.3)	
Asset Index						
1st quintile	507(25.3)	1500(74.7)	0.002	881(67.2)	430(32.8)	0.004
2nd quintile	510(23.4)	1668(76.6)		1017(70.6)	423(29.4)	
3rd quintile	607(21.5)	2217(78.5)		1305(70.7)	541(29.3)	
4th quintile	420(21.0)	1583(79.0)		875(67.0)	430(32.9)	
5^th ^quintile	738(24.3)	2299(75.7)		1042(72.7)	392(27.3)	
Mother Education						
None	2000(21.0)	7510(79.0)	<0.001	4354(68.1)	2042(31.9)	<0.001
Some education	782(30.8)	1757(69.2)		766(81.5)	174(18.5)	
Province						
Ouagadougou	184(27.0)	497(73.0)	<0.001	213(77.2)	63(22.8)	<0.001
Boucle de mouhoun	89(9.7)	827(90.3)		241(40.0)	362(60.0)	
Centre(sans Ouaga)	55(16.1)	287(83.9)		166(75.1)	55(24.9)	
Centre-sud	233(31.4)	510(68.6)		397(85.4)	68(14.6)	
Plateau central	162(15.5)	886(84.5)		469(71.6)	186(28.4)	
Centre-Est	102(12.3)	726(87.7)		324(63.6)	185(36.3)	
Centre-Nord	105(11.2)	836(88.8)		377(65.0)	203(35.0)	
Centre-Ouest	721(58.0)	522(42.0)		669(86.8)	102(13.2)	
Est	228(30.9)	510(69.1)		386(81.6)	87(18.4)	
Nord	150(18.6)	656(81.4)		343(70.0)	147(30.0)	
Cascades	172(19.4)	716(80.6)		352(66.7)	176(33.3)	
Hauts bassins	147(12.9)	989(87.1)		392(62.7)	233(37.3)	
Sahel	148(20.6)	569(79.4)		298(61.4)	187(38.6)	
Sud-Ouest	286(28.0)	736(72.0)		493(75.3)	162(24.7)	
Ethnicity						
Bobo	65(18.0)	296(82.0)	<0.001	117(58.8)	82(41.2)	<0.001
Dioula	95(12.2)	684(87.8)		233(51.7)	218(48.3)	
Fulfulde/Peul	115(17.4)	544(82.6)		246(58.2)	177(41.8)	
Gourmantche	218(32.4)	454(67.6)		360(82.4)	77(17.6)	
Gourounsi	284(52.5)	257(47.5)		282(89.5)	33(10.5)	
Lobi	313(30.0)	729(70.0)		502(75.1)	166(24.8)	
Mossi	1347(21.1)	5021(78.8)		2717(70.9)	1114(29.1)	
Senoufo	103(15.1)	580(84.9)		258(62.5)	155(37.5)	
Touareg/Bella	75(58.6)	53(41.4)		72(80.0)	18(20.0)	
Bissa	70(15.0)	397(85.0)		189(65.6)	99(34.4)	
Other	94(28.8)	232(71.2)		133(64.6)	73(35.4)	
Total	2782(23.1)	9267(76.9)		5120(69.8)	2216(30.2)	

	MFGM (Mean and Std. dev.)			Daughter FGM (Mean and Std. dev.)		

	No	Yes	p-value	No	Yes	p-value
Current age in year	26.5(10.0)	29.6(9.7)	<0.001	31.5(8.1)	38.0(7.2)	<0.001
Daughter age at FGM(years)					3.4(3.1)	

* RHCD Responsibility for health care decisions

Chi-square test was used for categorical data and Mann-Whitney test for continuous data.

### Data management and analysis

Data analysis was undertaken using SPSS Version 15 and STATA 10.0. Associations between potential risk factors and the prevalence of FGM were explored using two dichotomous outcome measures: i) whether a woman herself had had FGM; ii) whether she had one or more daughters with FGM; using chi-square, t-tests and Mann Whitney U test as appropriate. Logistic regression modeling was used to investigate social, demographic and economic risk factors associated with FGM and formal tests for interaction were undertaken.

Four models one for each of the main religious groups were undertaken for the logistic regression modeling, these were Protestant, Catholic, Muslim and traditional or animist. The following items were included: demographic variables: age (three 10 year age groups (15-24, 25-34, 35-49), urban versus rural living, region, ethnicity and religion), social variables: (any versus no education and responsibility for health care decisions (RHCD*)), and household economic status (asset index (divided into quintiles)). Tests for interaction between religion and each of the selected characteristics were also performed. Since the test for interaction between religion and education proved significant, subsequent analysis was stratified by religion (Table 2).

**Table 2:** Adjusted Odd ratio of Female Genital Mutilation (FGM) in Burkina Faso stratified by religion for various respondent characteristics and 95% Confidence interval (DHS 2003)

Religion	Protestant	Catholic	Muslim	Animist/trandit-ionalist
	**OR & 95%CI**	**OR & 95%CI**	**OR & 95%CI**	**OR & 95% CI**

Asset Index				
1st quintile	Reference	Reference	Reference	Reference
2^nd ^quintile	0.80(0.40, 1.40)	1.20(0.90, 1.60)	1.30(1.00, 1.70)	1.30(0.90, 1.70)
3rd quintile	0.90(0.50, 1.90)	1.40(1.00, 1.90)	1.20(1.00, 1.60)	1.50(1.00, 2.20)
4th quintile	1.20(0.50, 2.60)	1.50(1.10, 2.20)	1.10(0.80, 1.40)	0.90(0.50, 1.50)
5^th ^quintile	2.20(0.90, 5.20)	1.40(0.90, 2.10)	1.20(0.90, 1.60)	0.90(0.30, 2.20)
Age of respondent (years)				
15-24	Reference	Reference	Reference	Reference
25-34	0.60(0.40, 1.10)	0.50(0.30, 0.60)	0.50(0.40, 0.60)	0.40(0.30, 0.60)
35+	0.60(0.40, 1.00)	0.70(0.60, 0.90)	0.70(0.60, 0.80)	0.70(0.50, 1.00)
Place of residence				
urban	0.60(0.30, 1.20)	0.70(0.50, 1.10)	1.10(0.90, 1.50)	0.70(0.30, 1.80)
rural	reference	reference	reference	reference
Mother Education				
None	Reference	reference	reference	reference
Some education	0.70(0.60, 0.90)	0.70(0.60, 0.90)	0.80(0.70, 1.00)	2.00(0.90, 4.30)
Final say on health care				
Respondent alone	3.00(1.50, 5.90)	1.30(1.00, 1.80)	1.40(1.10, 1.90)	1.30(0.70, 2.40)
Respondent and husband	2.90(1.30, 6.60)	1.60(1.10, 2.40)	2.30(1.60, 3.40)	1.50(0.80, 2.70)
Husband/partner	1.50(0.90, 2.60)	1.50(1.20, 1.90)	1.50(1.20, 1.80)	0.90(0.60, 1.40)
Someone else	reference	reference	reference	reference
Province				
Ouagadougou	8.70(3.70, 20.5)	6.70(4.50, 10.1)	3.90(2.70, 5.80)	
Boucle de mouhoun	11.7(3.00, 46.1)	18.0(9.40, 34.5)	7.40(5.10, 10.7)	7.40(2.00, 27.0)
Centre(sans Ouaga)	9.10(2.40, 34.1)	10.5(6.50, 17.1)	4.10(2.40, 6.90)	8.70(0.90, 81.8)
Centre-sud	5.20(2.30, 11.5)	6.30(4.30, 9.10)	1.40(1.00, 1.90)	4.30(2.00, 9.30)
Plateau central	25.4(9.50, 67.8)	9.20(6.30, 13.3)	5.80(4.10, 8.20)	4.70(2.20, 10.1)
Centre-Est	14.8(4.10, 53.4)	32.6(19.0, 55.7)	4.00(2.80, 5.80)	19.1(5.20, 69.9)
Centre-Nord	14.1(4.70, 42.2)	35.9(19.0, 68.0)	4.20(3.00, 5.80)	31.2(15.2, 63.9)
Centre-Ouest	Reference	Reference	reference	reference
Est	13.2(3.40, 51.3)	18.5(9.80, 35.1)	2.30(1.30, 3.90)	7.00(2.00, 24.7)
Nord	2.80(0.90, 8.70)	6.80(3.90, 11.8)	3.30(2.50, 4.40)	7.10(2.70, 18.8)
Cascades	1.50(0.30, 7.10)	4.80(2.50, 9.40)	4.30(2.90, 6.20)	1.60(0.50, 4.60)
Hauts basins	12.2(3.90, 37.7)	9.40(5.90, 14.8)	7.60(5.50, 10.6)	2.60(0.40, 16.5)
Sahel	126.4(11.7, 136.7)	18.8(1.90, 183.9)	3.70(2.70, 5.20)	
Sud-Ouest	5.70(1.20, 27.3)	6.20(3.20, 11.9)	3.90(1.80, 8.60)	3.60(1.50, 8.90)
Ethnicity				
Bobo	1.50(0.30, 6.30)	3.60(1.70, 7.50)	1.80(1.10, 3.10)	1.90(0.30, 10.0)
Dioula	17.0(3.00, 97.8)	3.40(1.60, 7.40)	3.50(2.30, 5.20)	1.10(0.30, 3.80)
Fulfulde/Peul		1.50(0.20, 10.4)	3.20(2.30, 4.40)	
Gourmantche	1.30(0.30, 6.30)	0.70(0.30, 1.40)	1.90(1.10, 3.50)	0.20(0.10, 1.00)
Gourounsi	5.40(1.80, 16.1)	1.90(1.00, 3.50)	2.70(1.60, 4.80)	0.80(0.30, 2.60)
Lobi	3.80(0.70, 20.6)	1.90(0.90, 4.10)	2.30(0.90, 6.10)	0.70(0.30, 2.10)
Mossi	2.20(0.80, 6.00)	2.20(1.30, 3.90)	3.90(2.90, 5.30)	0.30(0.10, 0.90)
Senoufo	23.6(2.30, 237.4)	6.50(2.50, 16.6)	3.00(2.00, 4.60)	3.70(1.20, 12.1)
Touareg/Bella	Reference	reference	reference	reference
Bissa	2.80(0.70, 10.9)	1.00(0.50, 2.30)	5.40(3.30, 8.80)	0.30(0.10, 1.70)

## Results

Response rates were over 94% for individual women and over 98% at the household level. The resulting sample was representative of the underlying populations of the different regions of Burkina Faso [13]. Between June and December 2003, interviews were conducted in 9,097 households, with 12,049 women aged between 15-49 years and 3,065 men aged 15-59 years. Data collected were representative at the national level; at the urban/rural level and at the level of each administrative region when tested using a number of socio-demographic and household indicators [10].

Table 1 illustrates the bivariate results and shows that overall seventy seven percent (9267) of women had undergone FGM. Seven thousand three hundred and thirty six women (7336) reported that they had daughters of whom 2216 (30.2%) reported that they had a daughter who had undergone FGM and 334 (4.5%) reported that they intended that their daughter should have it.

A number of factors were significantly associated with FGM among respondents and their daughters as shown in Table 1. For both outcomes (i.e. women or daughter FGM status), there were significant consistent associations for several correlates of FGM. The results showed that age, religion, place of residence, household socio-economic status (health or wealth Index), maternal education, place of residence, province of residence, and ethnicity were all significantly related to the two outcomes (p < 0.01). As expected, FGM was more prevalent among older respondents (35-49 years old) and among their daughters. The proportion of women and daughters with FGM was higher amongst Muslim women. Women from rural areas were also significantly more likely to have undergone FGM as were their daughters compared to women from urban areas. There were significant differences between province of residence with respect to the two outcome variables with for example, higher prevalence of FGM in the province of Bouche de Mouhoun and a higher prevalence of FGM amongst women from the Dioula ethnic group and their daughters.

Higher prevalence of FGM was also found among respondents with no education and their daughters.

There was an inverse U-shape association between FGM and the wealth index for both outcomes - lower rates of FGM were found in middle income women and their daughters, compared to either higher or lower income women. This implies that the two extremes of the asset index (i.e. both poorer and richer household) are associated with higher risk of FGM. This non linear relationship between FGM and asset index is nonlinear, complex and begs an explanation.

When tested, significant interaction was found between religion and education. (Interaction term OR 1.14 (95%% CI 1.09-1.19 p < 0.0001). Multivariate regression models were therefore undertaken for the four different religious groups (See Table 2).

Table 2 indicates the separate results for each religious group. Household socio-economic status was not associated with FGM in the individual models for religious groups. For both Catholics and Protestants having some education reduced the likelihood of FGM. For all religious groups, there was a higher likelihood of FGM when either the respondent alone or the respondent and her husband together had responsibility for health care decisions For women who were Muslims or who practised a traditional or animist religion, there were strong associations with age group, although importantly none with education. Again, younger women (24-34 age groups) and those from specific regions and ethnicities were less likely to report themselves as having had FGM. Muslim women were also more likely to report themselves as having had FGM if they or their husband/partner had responsibility for healthcare decisions.

In summary, for most religious groups, younger age, responsibility for health care decisions lying outside the household (e.g with community leaders), living in a particular region (Centre-Ouest) or coming from a particular ethnicity (Touareg/Bella) were all associated with a reduced likelihood of undergoing FGM. And for all groups there was no difference between those living in urban versus rural areas.

However religion appeared to be an important factor for FGM, affecting the pattern of additional associations. For example education was associated with a reduced likelihood of undergoing FGM for both Protestant and Catholic women but not for Muslim women. And there was a complex relationship between household assets and FGM. However, when the analysis was stratified by religion, some categories particularly the different provincial or ethnic groups have a small sample. This means that findings should be interpreted cautiously.

Table 3 shows that after multiple adjustments, factors associated with a statistically significant decreased likelihood of FGM for women's daughters were higher socio-economic status: 5^th ^quintile OR 0.75 (95%% CI 0.58-0.97), younger (24-34 year old)/older - age of the mother and education.

**Table 3:** Adjusted Odd ratio and 95% CI of daughter's Female Genital Mutilation (FGM) in Burkina Faso (DHS 2003)

OR & 95%CI for daughter's FGM			
Asset Index		Religion	
1st quintile	Reference	Catholic	0.61(0.51, 0.72)
2^nd ^quintile	0.94(0.78, 1.13)	Protestant	0.46(0.33, 0.64)
3rd quintile	0.91(0.76, 1.09)	Trad./Animist	0.76(0.62, 0.93)
4th quintile	0.95(0.78, 1.16)	Muslim	Reference
5^th ^quintile	0.75(0.58, 0.97)		
Age of respondent (years)			
15-24	reference		
25-34	0.08(0.06, 0.32)		
35+	0.28(0.24, 0.32)		
Place of residence			
urban	0.90(0.72, 1.12)		
rural	reference		
Mother Education			
None	Reference		
Some education	0.67(0.54, 0.83)		
Final say on health care			
Respondent alone	0.95(0.72, 1.26)		
Respondent and husband	0.91(0.68, 1.23)		
Husband/partner	0.80(0.63, 1.02)		
Someone else	reference		
Province			
Ouagadougou	2.88(1.90, 4.36)		
Boucle de mouhoun	10.3(7.42, 14.3)		
Centre(sans Ouaga)	1.87(1.24, 2.81)		
Centre-sud	1.13(0.80, 1.61)		
Plateau central	2.35(1.74, 3.17)		
Centre-Est	4.01(2.86, 5.60)		
Centre-Nord	3.46(2.55, 4.69)		
Centre-Ouest	Reference		
Est	2.71(1.65, 4.45)		
Nord	2.73(1.99, 3.76)		
Cascades	2.81(1.90, 4.15)		
Hauts basins	4.79(3.48, 6.58)		
Sahel	4.10(2.87, 5.86)		
Sud-Ouest	2.06(1.22, 3.47)		
Ethnicity			
Bobo	1.19(0.76, 1.85)		
Dioula	1.55(1.07, 2.24)		
Fulfulde/Peul	1.75(1.22, 2.49)		
Gourmantche	0.82(0.48, 1.38)		
Gourounsi	0.85(0.51, 1.40)		
Lobi	1.42(0.83, 2.42)		
Mossi	1.35(1.00, 1.83)		
Senoufo	1.83(1.22, 2.77)		
Touareg/Bella	Reference		
Bissa	1.24(0.81, 1.92)		

## Discussion

### Overall findings

Age and religion were the most significant demographic variables associated with the risk of FGM in Burkina Faso. As age increased, the proportion of respondents and their daughters who had undergone FGM increased.

We found that FGM was strongly associated with religion. And our findings indicate that the pattern of associations with other variables was affected by women's religion: so that for Christian women, education appeared to have a protective effect, but this was not the case for Muslim women or for women who followed a traditional or animist religious tradition. Perhaps surprisingly, women taking responsibility for their own health care decisions were more likely to have had FGM. There were substantial variations between different regions of Burkina Faso and between different ethnic groups, although regional differences appeared greater than ethnic differences. Household asset index had varying associations depending on religion, with some evidence that in general, women in the middle asset quintiles were more likely to report themselves as having had FGM.

Returning to the relationship between women's own experience of FGM and that of their daughters we found that the difference between the percentage of younger and older women whose daughters had undergone FGM was much greater than the difference between the percentage of respondents who had undergone FGM themselves (Table 1). This could represent an important change in cultural mores with decreased use of FGM, whereby younger women are less willing to have their daughters undergo FGM. This offers possible hope that the practice may be reducing over time. However, this finding could also represent a cohort effect - younger women have younger children who are not yet at risk. We found a large difference in Burkina Faso in the risk of FGM between provinces and ethnicity with some provinces and ethnic groups having a higher risk of FGM. It is worth investigating these findings further as spatial variation and ethnicity may be indicative of an association of place of residence with FGM practice as a proxy for social norms.

### Strengths and weaknesses

The strength of this work is that it is based on a large well-conducted representative survey with a high response rate. Interviewers were trained; questions were standardized and data management procedures were exemplary [12].

The survey involved taking account of a large number of different factors including regions, ethnicities and others, but it is possible that some important factors particularly relevant for Burkina Faso have been missed. In addition, the topic area constitutes a complex social and policy issue affecting women's most intimate views of themselves and their lives. As with similar studies of this kind, we were dependent on women's self-reports to trained interviewers and cannot account for social acceptability bias which may have distorted findings, in particular involving selective under-reporting of FGM. There is likely to be a strong social pressure to conform, because of fear of social criticism, or pressure and disapproval from elders [16], which, coupled with the illegality of FGM in Burkina Faso may mean that individuals may have felt uncomfortable or unsure discussing or reporting FGM to strangers. However, notwithstanding, reported rates of FGM are high overall and appreciable even amongst daughters.

The survey was cross sectional. This may mean that we will have missed important cohort effects, for example in relation to FGM amongst daughters of younger women, although these would be of interest because of implications for future patterns and policy. For example, the difference between the percentage of younger and older participants whose daughters had undergone FGM was much greater than the difference between the percentages of respondents who had had FGM themselves (see Table 1). Whilst this could represent a change, whereby younger women are less willing to let their daughters have FGM, it could also represent the fact that younger women have younger children, who are not yet at risk.

### Implications for policy practice and research

There is already strong political opposition to the practice of FGM and consequently, it is internationally recognized as violation of human rights and an illegal activity in many countries. Burkina Faso is one of 16 African states which have outlawed FGM. Legislation was passed in 1996 with fines of up to 900,000 CFA (US $1,800) and prison sentences of up to three years for undertaking FGM. However making it illegal may have hampered women's reporting and seeking of safe medical treatment and of remedial measures where these are needed. Given the very personal effects of FGM and the contentious cultural mores associated with the practices of FGM, it would be valuable to review the law pertaining to FGM in Burkina Faso to identify any differences the law may be making. Religious links are clearly important, and although no religion prescribes the practice, policies which actively encourage engagement of religious leaders in its refutation would be beneficial.

As far as practical intervention is concerned, some strategies in Kenya have advocated alternative rites with the aim of involving communities alongside enforcing legislation.^1 ^And in Mali a programme of activities has been undertaken involving educating the women practitioners of FGM and giving them alternative skills for earning a living and spreading information to reduce public demand [17].

It has been suggested that FGM is a social convention in a country such as Nigeria [15] and that modernisation has little effect. However our results suggest that in the context of Burkina Faso, the education that modernization brings may benefit women from other religions more than Muslim women. Increasing assets and increasing women's responsibility for their own healthcare decision may have unexpected effects in increasing access to FGM, but there may be important benefits to be gained from projects which encourage women to work across and between different regions and ethnicities capitalizing on the geographical and cultural variations within Burkina Faso which exist.

In relation to promoting changing amongst populations of African communities living in countries such as the UK and Australia, the public health approach has tended to involve a confidential and supportive health service for women alongside a community education approach which is intended to raise awareness, changing attitudes to FGM and ultimately stop further FGM being carried out in the current country or country of origin.

Research should focus on cultural beliefs, reasons for ethnic, regional, religious and socio-economic differences in rates, on the optimal content of educational programmes, and methods for involving religious leaders. Longitudinal data would enable better tracking of practice through families.

## Conclusions

Although younger women with some education and those from specific groups, regions and religions are less likely to have had FGM it is still extremely common in Burkina Faso. Deep cultural issues and strongly personally held beliefs which are not simple to predict or quantify are likely to be involved in the perpetuation of FGM and there are no simple policy answers.

Policy initiatives should capitalize on our findings on the differences between religious groups. For Christian women, policy to reduce FGM should focus on education; for Muslim women and those from traditional religions, most benefits are likely to be gained from working with religious groups and leaders. Ideally policy should also be sensitive to the diversity and strength of women's beliefs and focus on ensuring that women are empowered to make their own decisions about FGM based on their own fully informed choices. However for many children, decisions are made whist they are still too young to participate in decisions and wider policy and cultural change is vital. Perhaps the more efficient and long lasting solution to eradicating the practice of FGM in Burkina Faso might be a global effort by the government and its development partners to make significant progress in the areas of employment, poverty reduction and literacy as well as a concerted effort to encourage repudiation of FGM and support for change by religious leaders.

## Competing interests

The authors declare that they have no competing interests.

## Authors' contributions

B K: Conception and design. Literature review. Data analysis and interpretation. Drafting the article. Critical revisions for important intellectual content. Approval of final article for submission. N-B K: Conception and design. Literature review. Data analysis and interpretation. Interpretation of results. Drafting the article. Critical revisions for important intellectual content. Approval of final article for submission. D C: Interpretation of results. Critical revisions for important intellectual content. A C: Interpretation of results. Critical revisions for important intellectual content. All authors read and approved the final manuscript.

## End note

*RHCD Categories included: Respondent alone; Respondent and husband; Husband/partner alone, Other e.g community leader
